# The Sydney Diabetes Prevention Program: A community-based translational study

**DOI:** 10.1186/1471-2458-10-328

**Published:** 2010-06-10

**Authors:** Stephen Colagiuri, Philip Vita, Magnolia Cardona-Morrell, Maria Fiatarone Singh, Louise Farrell, Andrew Milat, Marion Haas, Adrian Bauman

**Affiliations:** 1 Boden Institute of Obesity, Nutrition and Exercise, University of Sydney, NSW, Australia; 2 Sydney South West Area Health Service, NSW, Australia; 3 Exercise, Health and Performance Faculty Research Group, Faculty of Health Sciences, and Sydney Medical School, University of Sydney, NSW, Australia; 4 NSW Department of Health, NSW, Australia; 5 University of Technology, NSW, Australia; 6 School of Public Health, University of Sydney, NSW, Australia

## Abstract

**Background:**

Type 2 diabetes is a major public health problem in Australia with prevalence increasing in parallel with increasing obesity. Prevention is an essential component of strategies to reduce the diabetes burden. There is strong and consistent evidence from randomised controlled trials that type 2 diabetes can be prevented or delayed through lifestyle modification which improves diet, increases physical activity and achieves weight loss in at risk people. The current challenge is to translate this evidence into routine community settings, determine feasible and effective ways of delivering the intervention and providing on-going support to sustain successful behavioural changes.

**Methods/Design:**

The Sydney Diabetes Prevention Program (SDPP) is a translational study which will be conducted in 1,550 participants aged 50-65 years (including 100 indigenous people aged 18 years and older) at high risk of future development of diabetes. Participants will be identified through a screening and recruitment program delivered through primary care and will be offered a community-based lifestyle modification intervention. The intervention comprises an initial individual session and three group sessions based on behaviour change principles and focuses on five goals: 5% weight loss, 210 min/week physical activity (aerobic and strength training exercise), limit dietary fat and saturated fat to less than 30% and 10% of energy intake respectively, and at least 15 g/1000 kcal dietary fibre. This is followed by 3-monthly contact with participants to review progress and offer ongoing lifestyle advice for 12 months. The effectiveness and costs of the program on diabetes-related risk factors will be evaluated. Main outcomes include changes in weight, physical activity, and dietary changes (fat, saturated fat and fibre intake). Secondary outcomes include changes in waist circumference, fasting plasma glucose, blood pressure, lipids, quality of life, psychological well being, medication use and health service utilization.

**Discussion:**

This translational study will ascertain the reach, feasibility, effectiveness and cost-effectiveness of a lifestyle modification program delivered in a community setting through primary health care. If demonstrated to be effective, it will result in recommendations for policy change and practical methods for a wider community program for preventing or delaying the onset of type 2 diabetes in high risk people.

## Background

The number of people developing type 2 diabetes is rising dramatically worldwide with 439 million cases projected by 2030 [1]. This trend is mirrored in Australia where the prevalence of diabetes has more than doubled between 1981 and 2000 [2]. Currently in Australia there are over 1 million people with type 2 diabetes and approximately 8 per 1,000 people aged 25 years and older develop diabetes each year [3]. The annual health care costs in Australia of type 2 diabetes have been estimated at $2.2 billion AUD [4].

The increasing prevalence of diabetes, the increase in modifiable risk factors for the disease (obesity, sedentary behaviour and poor nutritional choices), as well as the severe and costly complications which can be difficult to prevent and treat, mean that prevention is an important strategy for reducing the burden of diabetes.

There is strong and consistent evidence from randomised controlled trials that type 2 diabetes can be prevented or delayed through lifestyle modification interventions which aim to improve diet, increase physical activity and reduce weight in people at high risk of developing the disease. The Finnish [5] and US [6] studies achieved a 58% reduction in diabetes incidence through lifestyle modification while the Chinese Da Qing study [7] achieved a 40% reduction in type 2 diabetes. Both the Finnish [8] and Chinese studies [9] showed long-term persistence of reduced diabetes risk over 7 and 20 years respectively. Similar benefits have been shown in the Japanese [10] and Indian [11] diabetes prevention trials, suggesting the generalisability of these findings.

Diabetes prevention programs recognise the importance of a theoretically-grounded behavioural intervention as a core component of the lifestyle modification, although no single behavioural theory has dominated. The behavioural programs have focused on encouraging physical activity and dietary changes and included various components such as initial assessment, individualised goal setting, individual counseling, on-going support, regular assessment and feedback and monitoring of behaviours and outcomes throughout the study.

Small community-based programs have reported some success in modifying surrogate markers for diabetes through lifestyle intervention. The Greater Green Triangle Diabetes Prevention Project in Australia [12] and the Good Ageing in Lahti Region Lifestyle Implementation Trial in Finland [13] confirmed that short-term lifestyle modification programs can reduce risk factors for diabetes in primary care settings.

The current challenge is to translate this evidence into cost-effective large scale community-wide programs. There is increasing acknowledgement that the best way to do this is through studies which have an explicit focus on generalisation and feasibility and which report information on contextual variables such as representativeness, reach, implementation and adaption, costs and other outcomes important to policy makers [14]. Thus the current study employs a design which will not only provide data on intervention effectiveness, but also will examine contextual factors through process evaluation central to what has been called by some the 'science of delivery' [15].

The overall aim of this study is to assess the effectiveness of a community-based lifestyle modification program on modifiable risk factors for type 2 diabetes. Additional aims include assessing the feasibility of delivering the program in a primary health care setting; identifying determinants of the interventions which are associated with and predict a beneficial outcome; and the costs and cost-effectiveness of the program.

## Methods/Design

The SDPP is a translational study based on the active arm of international randomised controlled trials demonstrating the effectiveness of lifestyle modification interventions in reducing the incidence or delaying the onset of type 2 diabetes in high risk individuals.

### Setting

Communities in three Divisions of General Practice, one in metropolitan Sydney, one in a semi-rural area and the other in a rural area of NSW, Australia.

### Ethics

Ethics approval to conduct this trial has been granted by the Research Ethics Review Committee of the Sydney South West Area Health Service - Eastern Zone (ID Number X08-0053). Written informed consent is obtained by all participants prior to enrolment.

### Participants and recruitment

The Divisions of General Practice have recruited over 75 practices and 150 general practitioners (GPs) to participate in the study. This was done through expression of interest by letter and fax, information sessions and site visits. The main pre-requisite for inclusion was the practice having a computerised patient record system.

People aged 50-65 years (18 years and older in the indigenous subgroup) without known diabetes attending study general practices are approached to participate. A variety of methods are used to identify potential participants including opportunistic recruitment when the person attends the general practitioner for a routine consultation, using the practice's computer database to identify people in the target age range and sending a letter suggesting they attend for risk assessment, and local media advertising of the program.

Risk is assessed using the AUSDRISK tool, a questionnaire developed in Australia [16]. The questions focus on demographic and diabetes risk factors and include an objective assessment of waist circumference performed by trained research staff. The maximum AUSDRISK score is 38 and a score of ≥ 15 is considered high risk. A score of 15-19 is associated with a 1 in 7 chance and a score of ≥ 20 with a 1 in 3 chance of developing diabetes over the next 5 years [16]. A score of ≥ 12 is considered high risk in the indigenous subgroup.

A person with a risk score of ≥ 15 is required to undergo investigations to exclude prevalent diabetes. This initially involves measurement of capillary blood glucose in the general practitioner's surgery, followed by measurement of fasting plasma glucose and possibly an oral glucose tolerance test.

Study exclusion criteria include new or previously diagnosed diabetes, taking a hypoglycaemic medication in the past month, use of prescribed weight loss medication or a medical contraindication to participation in a physical activity program. Participants enter the study after written consent is obtained, eligibility criteria have been met and clearance is received from their general practitioner.

The study plans to recruit 1,550 high risk people into the lifestyle intervention program. Among these 100 will be Arabic-speaking, 100 will be Chinese-speaking and 100 will be indigenous people.

### Intervention - Lifestyle Modification Program

The five aims of the lifestyle modifications are:

1. At least 30 min/day of moderate to vigorous intensity physical activity, including aerobic exercise 3 or more days/week plus strength training at least twice/week (210 min/week total structured exercise)

2. Reduction in the intake of energy from total fat to less than 30%

3. Reduction in the intake of energy from saturated fat to less than 10%

4. Fibre intake of at least 15 g/1000 kcal

5. Achievement of a 5% reduction in body weight at 12 months.

In addition to the 210 min/week structured exercise goal, participants are encouraged to increase incidental physical activity in ways which would enhance both cardiovascular and musculoskeletal fitness.

These five goals are entirely concordant with the Finnish DPS (5), which was one of the most successful diabetes prevention trials. The physical activity goal, which has been modified slightly from the Finnish DPS is based on a review of the physical activity prescriptions utilised in relation to outcomes achieved in all of the successful trials of diabetes prevention (5,6,7,10,11), considerations of cost and feasibility in this translational setting, as well as other literature regarding modality, volume, and intensity of exercise required to improve metabolic risk and body composition in similar cohorts. Both the Finnish DPS and the US DPP included resistance training (strength training) in their supervised exercise sessions and is explicitly specified within the physical activity goal of the SDPP. Resistance training is an anabolic form of exercise, differing substantially from aerobic exercise in its ability to induce muscle hypertrophy and associated metabolic and functional changes [17,18]. It improves insulin sensitivity, glucose homeostasis, blood pressure, dyslipidaemia, markers of inflammation and catabolism, and visceral obesity, thus addressing the key metabolic abnormalities in adults at high risk of type 2 diabetes [19,20]. Importantly, resistance training (but not aerobic exercise) attenuates or prevents the loss of lean tissue (muscle and bone) accompanying weight loss diets such as those prescribed in this study [21].

The behavioural components are based on stages of change [22] and social cognitive theories [23]. The intervention is delivered by dedicated program lifestyle officers from a variety of health backgrounds including dietetics, nursing, psychology and exercise physiology. The lifestyle officers undergo specific training in health coaching, group program delivery and standardized data collection used for evaluation. The health coaching approach incorporates principles from self-management, removing psychological blocks to change and confidence [24].

**Figure 1:**
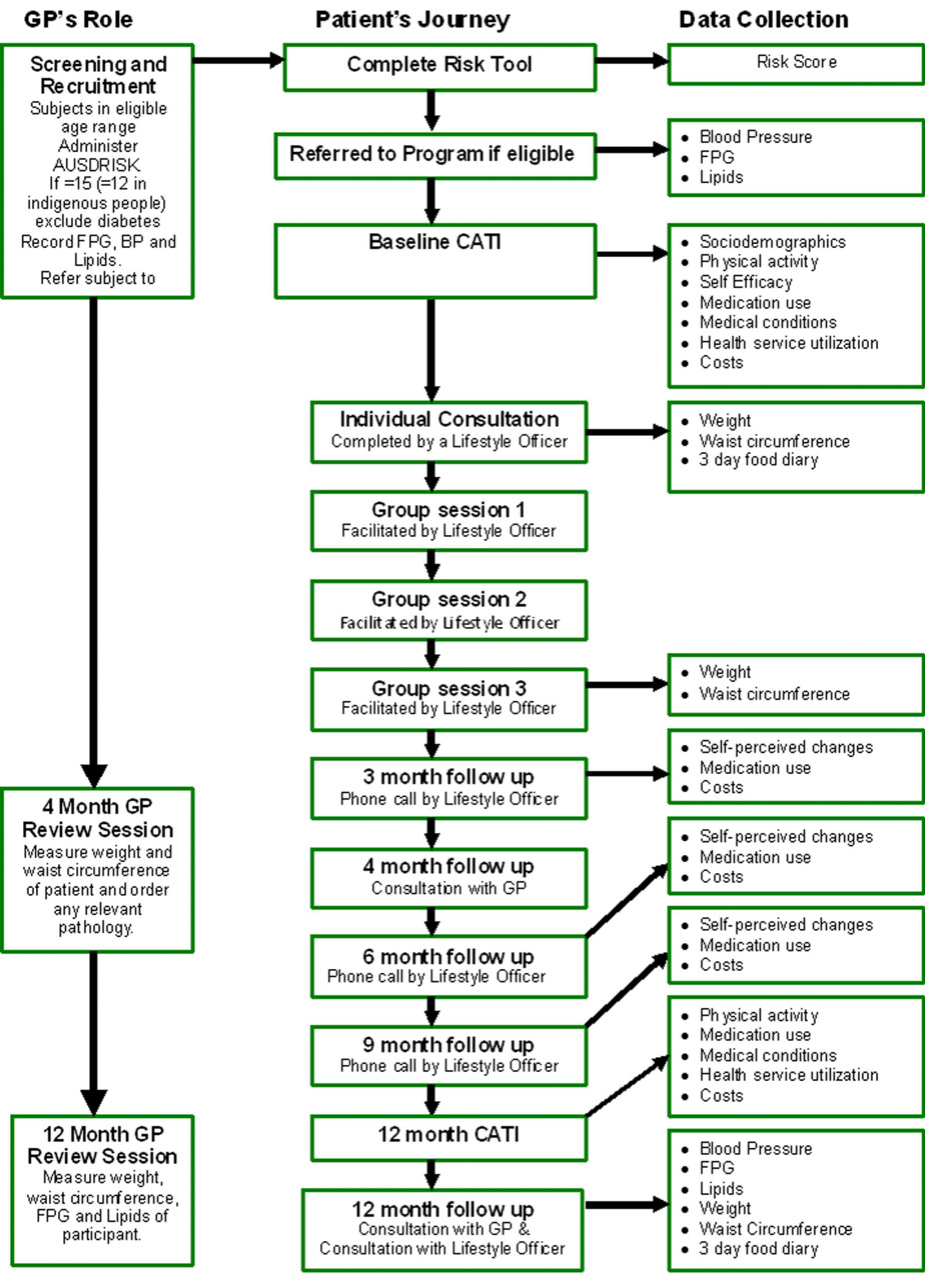
**Overview of the program and data collection points for evaluation**.

An overview of the program and the evaluation plan is shown in Figure 1.

High risk individuals agreeing to participate in the lifestyle modification program complete an initial computer-assisted telephone interview (CATI) survey. This survey includes socio-economic and demographic information, physical activity habits, quality of life, and self-efficacy, as well as recent health service utilisation and current medication use. Participants are then scheduled to attend an individual consultation with a lifestyle officer. At this consultation, the lifestyle officers measure height, weight and waist circumference using calibrated stadiometers, scales and tape measures, following a standardized anthropometric protocol as specified by the International Society for the Advancement of Kinanthropometry (ISAK) [25]. The individual consultation includes a general discussion about diabetes risk and prevention, an overview of the program, and uses motivational interviewing techniques to assist participants to set goals and develop tools to self-monitor. Following this session, arrangements are made for participants to attend three two-hour group programs held over a six to eight week period. Lifestyle officers conduct these group sessions of approximately 10 people, which cover theoretical, behavioural and practical aspects of diet and physical activity. The overall program motto is: "Eat better and move more". Those who are not able to or do not want to attend a group program are offered the option of three individual health coaching sessions by telephone, covering the same material. The intervention delivered to indigenous participants will be slightly modified to take account of cultural issues.

Follow up telephone calls are made by the lifestyle officers to each participant at 3, 6 and 9 months to enquire about progress, assist with behaviour change and offer participants additional support as required.

In addition, participants are provided with details of local community-based lifestyle programs which have been evaluated by the research staff and found to be consistent with the goals of the SDPP. Participants have the option of enrolling in such programs as one way to assist in achieving the SDPP physical activity and dietary goals.

At 12 months the CATI survey is repeated and participants undergo an individual assessment with the lifestyle officer and their general practitioner.

### Outcome measures and evaluation plan

The evaluation plan has three components:

1. Impact evaluation measures changes at 12 months in the key outcomes known to be associated with reduced diabetes incidence: weight loss; increase in moderate to vigorous physical activity (including both aerobic and strengthening activities); increase in dietary fibre consumption; decrease in fat consumption and decrease in saturated fat intake. Program and participant factors which predict these outcomes are assessed.

2. Process evaluation measures program reach, fidelity, satisfaction and knowledge of program delivery by staff, satisfaction of consumers, and identifies facilitators and barriers associated with program implementation and delivery.

3. Economic evaluation involves health system and individual perspectives and estimates implementation costs, costs per outcome and cost-effectiveness.

#### Impact Evaluation

Outcomes are assessed at baseline and 12 months by research staff, using a combination of in-person assessment and CATI questionnaires. Physical activity is assessed using the Physical Activity Scale for the Elderly (PASE), which has established reliability and validity [26]. Dietary intake (including total fat, saturated fat and fibre) is assessed by a self-completed 3-day non-weighed food record, which is analysed using Foodworks [27].

Weight, height, waist circumference and blood pressure are measured using standard methods. Blood is collected for measurement of fasting plasma glucose and lipids. Additional questions on socio-demographic characteristics, concomitant diseases and medications, smoking, alcohol, social support, self-efficacy for lifestyle change, and quality of life are asked at the baseline and 12 month CATI surveys.

Analyses of these data will determine whether the program goals are achieved by individuals, by the group as a whole and differentially by sub-groups (e.g. GP Division and gender). In addition, these data will be used to model the projected impact of the program beyond the 12-month intervention period.

#### Process Evaluation

This is a central component of the evaluation of this translational study in order to assess whether the program is implemented as planned, achieves population reach and whether it is feasible in the primary care field setting. Details of the process evaluation components are shown in Table 1.

**Table 1:** Process evaluation components in the Sydney Diabetes Prevention Program

Evaluation Component	Data source and format	Timeframe
Screening, participation and recruitment rates	Administrative documentation from Divisions of General Practice	Ongoing during recruitment
Program fidelity, program completion, intervention completed	Participants' database	Ongoing
Assessment of practice staff awareness and engagement with program	Telephone administered questionnaires to selected doctors and practice staff	Ongoing
Barriers to recruitment, and program delivery	In-depth-interviews and focus groups with practice staff	Ongoing
Challenges in program delivery and patient maintenance in program.	Focus group with lifestyle officers	Ongoing
Participants' barriers to attendance at group sessions	From participants via the Lifestyle Officers	Three months after group sessions completed
Number and type of organisations participating, perceived level of collaboration, barriers and success factors for community-based programs/services for physical activity and weight management	In-depth-interviews and focus groups with key stakeholders	Towards the end of the Program, when 12 month follow up data available

#### Economic Evaluation

The economic evaluation will adopt a health system perspective by measuring costs at Area Health Service, Government and general practice levels. It will also use a limited societal perspective by measuring direct costs to participants. In addition, the estimated costs of a state-wide rollout of the SDPP will be reported. The base cost year will be 2008. No discounting will be applied as this is a 12-month program including intervention and follow-up.

The economic evaluation will compare the cost of program implementation with its intermediate outcomes. The results of the study will be used to build a model of future costs and effects beyond the study period using Australian population and risk factor data [28-32]. Information to be collected about resource use will include program expenses (e.g. cost of screening and delivering the intervention), individual level and health system expenditure data. These will include direct health-related costs (visits to health professionals, hospitalisation, medication use etc) and direct non-health costs (gym subscription, exercise equipment, etc.) relevant to diabetes prevention, and indirect costs (e.g. sick days). This information will allow estimation of the cost per estimated case of diabetes prevented and cost per outcome (e.g. cost per kg weight loss; cost per change in additional time of physical activity, etc). Modelling will project these calculations beyond the study period using lives saved, life-years saved and quality-adjusted life-years (QALYs) gained to calculate the benefits associated with this program.

#### Statistical methods

Analysis at 12 months will determine the extent that program goals were achieved, through overall and sub-group analysis. Primary outcomes which will be reported include weight loss (and 5% weight loss goal achievement), physical activity levels (in minutes per week of overall intentional activity, and sessions per week of strength training) and PASE scores, dietary measures (total energy/day, estimated fibre intake (gm/1,000 kcal), total fat intake (gm/day), fat as a proportion of total energy intake and saturated fat as a proportion of daily energy intake), and proportions of the cohort achieving 1,2,3,4, or all 5 goals. Logistic regression analysis will be used to examine factors associated with the achievement of program goals. Separate models will examine predictors of 5% weight loss, achievement of 210 min/day of physical activity, and achievement of dietary goals.

Missing data due to either refusal to respond to selected questions or withdrawal from the program before the endpoint will be dealt with by imputing values for the discrepant self-reports using data from "similar" participants or using last observation carried forward (LOCF) techniques when appropriate.

A minimal sample size of 1,000 completed participants with baseline and 12 month data will provide 90% power to detect small differences in primary outcomes effects at the 5% level of confidence for the whole group. Specifically, we will be able to detect:

• 5% weight loss

• increase of 2.5 g of fibre/1,000 kcal

• 3.4% reduction of fat intake as a percentage of total energy

• 2.7% reduction of saturated fat intake as a percentage of total fat intake

• 11.7 min/wk of increased physical activity.

## Discussion

Lifestyle modification has been shown to effectively reduce the risk of incident diabetes in randomised controlled trials. The main challenge is to translate this evidence into a routine community-wide setting and provide a feasible, effective and cost-effective intervention. The current study is designed to address these presently unanswered public health and policy-relevant questions.

The information gathered in this study will be of direct relevance to the design, implementation and affordability of community-wide diabetes prevention programs throughout Australia and other countries with a well-developed health care system. The key questions which can be addressed by the SDPP relate to the delivery, effects, costs and structure of community-based lifestyle modification programs, including key barriers and facilitators and key determinants of process and impact outcomes. Importantly, the study should provide a better understanding of the interplay of these factors in real-world settings in metropolitan, regional and rural settings across populations of differing socioeconomic status and ethnic background and by doing so increase the generalisability of the findings. Answers to these issues are of direct relevance to future local, national, and international evidence-based policy and practice with regard to prevention of diabetes and lifestyle-related chronic diseases. The study will also provide an understanding of the preferences of participants for lifestyle-related strategies and programs, which will also be relevant to the design of future strategies and programs.

## List of abbreviations used

AUD: Australian Dollar; BP: blood pressure; CATI: Computer-assisted telephone interview; DPP: Diabetes Prevention Program; DPS: Diabetes Prevention Study; FPG: fasting plasma glucose; GPs: General Practitioners; PASE: Physical Activity Scale for the Elderly; QALY: Quality Adjusted Life Year; SDPP: Sydney Diabetes Prevention Program.

## Competing interests

The authors declare that they have no competing interests.

## Authors' contributions

SC conceived of the study, and participated in its design, evaluation and implementation. PV participated in finalising the protocol and is responsible for the implementation of the study. MC-M participated in the design of the study evaluation and is performing the statistical analyses. MFS participated in the study design, its implementation and evaluation. LF participated in the design and implementation of the study evaluation. AM participated in the design, evaluation and implementation of the study. MH participated in the design and the general and economic evaluation of the study. AB participated in the study design and implementation and conceived and oversees the study evaluation. All authors made substantial contributions to the conception, design and writing of the manuscript and had the opportunity to critically review the manuscript during its development and all approved the final manuscript.

## Pre-publication history

The pre-publication history for this paper can be accessed here:

http://www.biomedcentral.com/1471-2458/10/328/prepub
